# Development of a Standardized Screening Rule for Tuberculosis in People Living with HIV in Resource-Constrained Settings: Individual Participant Data Meta-analysis of Observational Studies

**DOI:** 10.1371/journal.pmed.1000391

**Published:** 2011-01-18

**Authors:** Haileyesus Getahun, Wanitchaya Kittikraisak, Charles M. Heilig, Elizabeth L. Corbett, Helen Ayles, Kevin P. Cain, Alison D. Grant, Gavin J. Churchyard, Michael Kimerling, Sarita Shah, Stephen D. Lawn, Robin Wood, Gary Maartens, Reuben Granich, Anand A. Date, Jay K. Varma

**Affiliations:** 1 World Health Organization, Geneva, Switzerland; 2 Thailand Ministry of Public Health - U.S. Centers for Disease Control and Prevention Collaboration, Nonthaburi, Thailand; 3 United States Centers for Disease Control and Prevention, Atlanta, United States of America; 4 Department of Clinical Research, London School of Hygiene & Tropical Medicine, London, United Kingdom; 5 ZAMBART Project, University of Zambia Ridgeway Campus, Lusaka, Zambia; 6 Aurum Institute for Health Research, Johannesburg, South Africa; 7 Bill and Melinda Gates Foundation, Seattle, United States of America; 8 Albert Einstein College of Medicine, New York, United States of America; 9 Desmond Tutu HIV Center, University of Cape Town, South Africa; 10 Department of Medicine, University of Cape Town, South Africa; Harvard School of Public Health, United States of America

## Abstract

**Background:**

The World Health Organization recommends the screening of all people living with HIV for tuberculosis (TB) disease, followed by TB treatment, or isoniazid preventive therapy (IPT) when TB is excluded. However, the difficulty of reliably excluding TB disease has severely limited TB screening and IPT uptake in resource-limited settings. We conducted an individual participant data meta-analysis of primary studies, aiming to identify a sensitive TB screening rule.

**Methods and Findings:**

We identified 12 studies that had systematically collected sputum specimens regardless of signs or symptoms, at least one mycobacterial culture, clinical symptoms, and HIV and TB disease status. Bivariate random-effects meta-analysis and the hierarchical summary relative operating characteristic curves were used to evaluate the screening performance of all combinations of variables of interest. TB disease was diagnosed in 557 (5.8%) of 9,626 people living with HIV. The primary analysis included 8,148 people living with HIV who could be evaluated on five symptoms from nine of the 12 studies. The median age was 34 years. The best performing rule was the presence of any one of: current cough (any duration), fever, night sweats, or weight loss. The overall sensitivity of this rule was 78.9% (95% confidence interval [CI] 58.3%–90.9%) and specificity was 49.6% (95% CI 29.2%–70.1%). Its sensitivity increased to 90.1% (95% CI 76.3%–96.2%) among participants selected from clinical settings and to 88.0% (95% CI 76.1%–94.4%) among those who were not previously screened for TB. Negative predictive value was 97.7% (95% CI 97.4%–98.0%) and 90.0% (95% CI 88.6%–91.3%) at 5% and 20% prevalence of TB among people living with HIV, respectively. Abnormal chest radiographic findings increased the sensitivity of the rule by 11.7% (90.6% versus 78.9%) with a reduction of specificity by 10.7% (49.6% versus 38.9%).

**Conclusions:**

Absence of all of current cough, fever, night sweats, and weight loss can identify a subset of people living with HIV who have a very low probability of having TB disease. A simplified screening rule using any one of these symptoms can be used in resource-constrained settings to identify people living with HIV in need of further diagnostic assessment for TB. Use of this algorithm should result in earlier TB diagnosis and treatment, and should allow for substantial scale-up of IPT.

*Please see later in the article for the Editors' Summary*

## Abstract 2

Haileyesus Getahun and colleagues report the development of a simple, standardized tuberculosis (TB) screening rule for resource-constrained settings, to identify people living with HIV who need further investigation for TB disease.

## Editors' Summary

**Background:**

In 2009, 1.7 million people died from tuberculosis (TB)—equating to 4,700 deaths a day—including 380,000 people living with HIV. TB remains the most common cause of death in people living with HIV and compared to people without HIV, people living with HIV are more than 20 times more likely to develop TB. Furthermore, TB infection may occur at any stage of HIV disease and is often the initial presentation of underlying HIV infection. Without antiretroviral treatment, up to 50% of people living with HIV who are diagnosed with TB die during the 6–8 months of TB treatment.

Although antiretroviral treatment can reduce the incidence of TB both at the individual and population level, people living with HIV on antiretroviral treatment still have higher TB incidence rates and a higher risk of dying from TB. Therefore, the World Health Organization recommends regular screening for active TB disease in all people living with HIV, so those identified as having active TB disease can be provided with appropriate treatment, and isoniazid preventive therapy (to help mitigate TB morbidity, mortality, and transmission) can be given to vulnerable individuals who do not yet have active TB.

**Why Was This Study Done?:**

There is currently no internationally accepted evidence-based tool to screen for TB in people living with HIV—a serious gap given that the presenting signs and symptoms of TB in people living with HIV are different from those in people without HIV. Therefore, the researchers aimed to develop a simple, standardized TB screening rule for resource-constrained settings, on the basis of the best available evidence that would adequately distinguish between people living with HIV who are very unlikely to have TB from those who require further investigation for TB disease.

**What Did the Researchers Do and Find?:**

The researchers selected 12 studies that met their strict criteria, then asked the authors of these studies for primary data so that they could map individual-level data to identify five symptoms common to most studies. Using a statistical model, the researchers devised 23 screening rules derived from these five symptoms and used meta-analysis methods (bivariate random-effects meta-analysis) and the association of study-level and individual-level correlates (hierarchical summary relative operating characteristic curves) to evaluate the sensitivity and specificity of each tool used in each individual study.

The authors of the selected studies were able to provide data for 29,523 participants, of whom 10,057 were people living with HIV. The dataset included 9,626 people living with HIV who had TB screening and sputum culture performed, of which 8,148 individuals could be evaluated on the five symptoms of interest from nine of 12 studies. TB disease was diagnosed in 5.8% of people living with HIV and the best performing rule was the presence of any one of the following: current cough (any duration), fever, night sweats, or weight loss. The overall sensitivity of the rule was 78.9% and the specificity was 49.6%. However, the sensitivity of the rule increased to 90.1% among participants selected from clinical settings and to 88.0% among those who were not previously screened for TB.

**What Do These Findings Mean?:**

The results of this study suggest that in resource-constrained settings, the absence of current cough, fever, night sweats, and weight loss (all inclusive) can identify those people living with HIV who have a low probability of having TB disease. Furthermore, any one of these symptoms can be used in resource-constrained settings to identify people living with HIV who are in need of further diagnostic assessment for TB.

Despite the limitations of the methodology used in this study, until there are evidence-based and internationally recommended guidelines for the diagnosis and treatment of TB in people living with HIV, use of the algorithm developed and presented in this study could result in earlier TB diagnosis and treatment for people living with HIV and could help to substantially scale-up isoniazid preventive therapy.

**Additional Information:**

Please access these websites via the online version of this summary at http://dx.doi.org/10.1371/journal.pmed.1000391.

- The World Health Organization has information about TB in people living with HIV
- The US Centers for Disease Control and Prevention also provide information about TB and HIV coinfection
- The World Health Organization also has information about isoniazid preventative therapy
- The Stop TB Partnership's TB/HIV Working Group provide information about TB and HIV co-infection

## Introduction

By the end of 2009, an estimated 33 million people were living with HIV, the vast majority in sub-Saharan Africa and Asia. Tuberculosis (TB) remains the most common cause of death in people living with HIV. Compared to people without HIV, people living with HIV have a more than 20-fold increased risk of developing TB [1]. TB disease may occur at any stage of HIV disease and is frequently the first recognized presentation of underlying HIV infection [2],[3]. Without antiretroviral treatment (ART), up to 50% of people living with HIV who are diagnosed with TB die during the 6–8 mo of TB treatment [4]–[6]. This risk increases to 72%–98% among those with multi-drug (MDR) or extensively drug-resistant (XDR) TB [7],[8]. Although ART can reduce the incidence of TB both at individual [9] and population [10] levels, people living with HIV on ART still have higher TB incidence rates and a higher risk of dying from TB [11]. Routine TB screening offers the opportunity to diagnose and promptly treat TB disease, and to identify those without TB disease who may be eligible for TB preventive therapy [12]. The use of TB preventive therapy can reduce TB incidence and is therefore of considerable benefit to patients [13].

For these reasons, the World Health Organization (WHO) recommends regular screening for active TB disease of all people living with HIV and providing either treatment for active disease or isoniazid preventive therapy (IPT) to mitigate TB morbidity, mortality, and transmission [14]. However, in 2009, of the estimated 33 million people living with HIV, only 1.7 million (5%) were screened for TB, and about 85,000 (0.2%) were offered IPT [15]. Currently there is no internationally accepted evidence-based tool to screen for TB in people living with HIV. Several studies have shown that the presenting signs and symptoms of TB in people living with HIV are different from those in people without HIV to diagnose TB; for example, many people living with HIV who have culture-confirmed TB do not report having a prolonged cough, which is one of the standard TB screening questions used by national TB control programs globally [16]. Moreover, the most widely available TB diagnostic tests such as smear microscopy and chest radiography perform poorly among people living with HIV; because most people living with HIV and TB have either sputum acid-fast bacilli (AFB) smear negative pulmonary or extrapulmonary TB [17].

We conducted an individual participant data meta-analysis of published and unpublished studies to develop a simple, standardized TB screening rule for resource-constrained settings that will adequately separate patients into two groups: (1) those for whom TB is reliably excluded, and IPT and ART, if indicated, can be initiated; and (2) those who require further investigation for TB disease. We describe the results of this meta-analysis and propose an algorithm for TB screening among people living with HIV in resource-constrained settings.

## Methods

We proceeded through several steps. First, we prospectively enumerated criteria for studies to be included in our meta-analysis. Second, we searched for and selected studies that met these criteria. Third, we sought primary data from investigators and mapped individual-level data to common symptoms. Fourth, we identified five symptoms available in most studies and, within each study, computed the sensitivity and specificity of 23 screening rules derived from these five symptoms. Finally, we used meta-analysis methods to estimate the performance of all 23 rules, as well as the association of study-level and individual-level correlates with performance.

### Inclusion of Studies

We defined studies as being eligible for inclusion in this analysis if they met the following criteria: (1) collected sputum specimens from people living with HIV regardless of signs or symptoms; (2) used mycobacterial culture of at least one specimen to diagnose TB; and (3) collected data about signs and symptoms.

### Search Strategy and Selection of Studies

To identify studies eligible for the meta-analysis, we conducted a systematic literature review of studies related to TB screening among people living with HIV in June 2008 using PubMed and various combinations of the following keywords: “HIV,” “Tuberculosis,” “TB screening,” “Smear-negative TB,” “Sputum negative TB,” “TB case finding,” “Intensified TB case finding,” “Isoniazid prevention treatment, trial or therapy.” We also searched for abstracts presented at conferences organized by the International Union Against TB and Lung Diseases and the International AIDS Society between 2000–2008. No language restriction was placed on the search. We reviewed all retrieved titles and abstracts for relevance to the topic. The reference lists of retrieved studies were also reviewed to identify further studies that meet the eligibility criteria. In addition, recognized experts in the field were contacted to identify studies that were not available (e.g., unpublished) in the initial electronic search. Studies that involve concomitant HIV testing and mycobacterial culture on all patients are resource intensive and challenging to implement in countries with a high burden of TB and HIV. Therefore, we believe it is unlikely that eligible studies would have been completed but missed by our search strategy.

**Figure 1:**
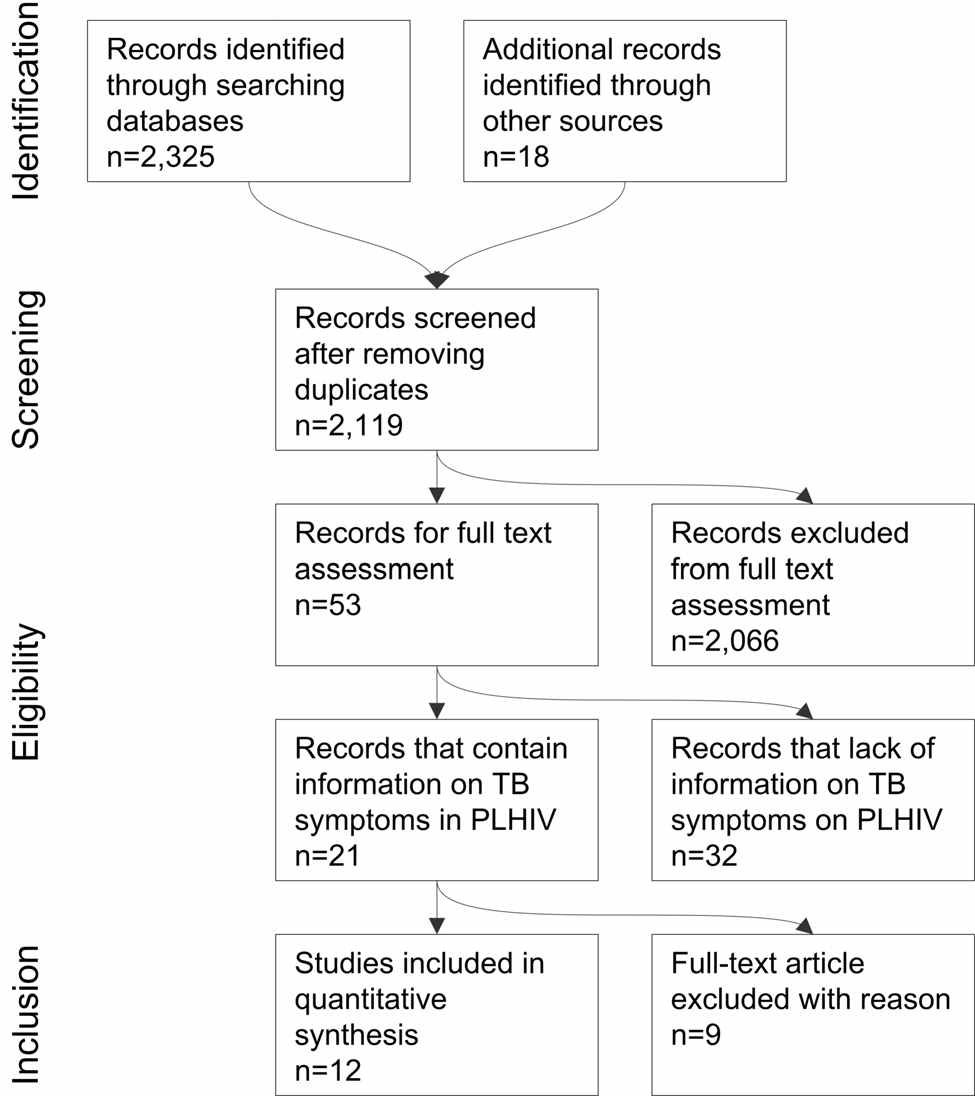
Search strategy and studies included in the meta-analysis (PRISMA flow diagram).

We found 2,119 publications and reviewed all their abstracts. Using the criteria above, we selected 53 for review of the full text. Twenty-one articles included information on signs and symptoms for TB screening in people living with HIV. A total of 14 studies (six published and eight unpublished at the time of the search) met the inclusion criteria of our meta-analysis (Figure 1). The corresponding authors or principal investigators were contacted for all 14 studies to confirm that their studies met all the eligibility criteria. One unpublished dataset was excluded for not meeting the inclusion criteria after verification with the principal investigator, and another one was excluded because the investigators could not submit the data within the agreed timeframe. A total of 12 investigators (representing six published and six unpublished studies at the time of the search) provided de-identified individual patient data for inclusion in the primary meta-analysis within an agreed time framework (Table 1) [18]–[29]. In November 2010, immediately preceding manuscript publication, we re-ran the search strategy again to look for additional studies that were reported since the initial search and should have been included in the meta-analysis. The search found seven eligible studies, of which all except one [30] were included in our meta-analysis as unpublished datasets.

**Table 1:** Summary of studies included in the meta-analysis.

Reference	Study Population Characteristics (Setting, Continent, n Samples, Culture Method Used)	Sample Size	PLTB/PLHIV^a^ (%)	PLTB/PLHIV (%) with Data on the Five Symptoms
Ayles et al. 2009	Adults of more than 15 y of age sampled from one rural and one urban communities in Zambia (community, sub-Saharan Africa, 1 LJ and MGIT).	8,044	43/2,253 (1.9)	41/2,145 (1.9)
Corbett et al. 2010^b^	Random sample of adults in 46 previously enumerated neighbourhoods in the high density suburbs of Harare, Zimbabwe (community, sub-Saharan Africa, 3 LJ).	10,079	31/1,841 (1.7)	31/1,834 (1.7)
Cain et al. 2010	PLHIV from 8 outpatient clinics in Cambodia, Thailand, and Vietnam who were enrolled regardless of signs or symptoms suggestive of TB (clinical, Southeast Asia, 3 MGIT and LJ).	1,748	267/1,724 (15.5)	267/1,721 (15.5)
Day et al. 2006	Employees of a gold mining company first attending the TB preventive therapy clinic in South Africa (miners, sub-Saharan Africa, 2 LJ).	1,093	32/991 (3.2)	0/0 (–)
Corbett et al. 2007^b^	Employees of 22 small and medium-sized enterprises in Zimbabwe (community, sub-Saharan Africa, 3 LJ).	4,668	3/797 (0.4)	3/797 (0.4)
Lewis et al. 2009^b^	All consenting employees of a gold mining indusry who undergo annual medical examinations in an occupational health centre in South Africa (miners, sub-Saharan Africa, 2 LJ).	1,955	18/560 (3.2)	18/560 (3.2)
Shah et al. 2009	All newly diagnosed HIV-positive clients of at least 18 y old from the VCT Clinic in a large referral hospital in Addis Ababa, Ethiopia (clinical, sub-Saharan Africa, 1 LJ).	453	27/427 (6.3)	22/357 (6.2)
Kimerling et al. 2002	PLHIV of at least 15 y of age and enrolled in an HIV home-based care service in Phnom Penh, Cambodia (community, Southeast Asia, 1 LJ).	441	36/393 (9.2)	36/393 (9.2)
Lawn et al. 2009	PLHIV with more than 18 y of age who were referred to a community-based ART service in South Africa (clinical, sub-Saharan Africa, 2–4 MGIT).	235	58/226 (25.7)	57/218 (26.1)
Wood et al. 2007	Randomly sampled and consenting adults living in shacks in a high-density residential area in South Africa (community, Sub-Saharan Africa, 4 MGIT).	174	12/163 (7.4)	0/0 (–)
Mohammed et al. 2004	PLHIV with WHO clinical stage 3 or 4 disease referred for possible participation in a trial of TB-preventive therapy in 3 hospital-based adult HIV clinics in South Africa (clinical, sub-Saharan Africa, 1 BACTEC).	129	10/128 (7.8)	0/0 (–)
Chheng et al. 2008	All consenting participants of more than 19 y old who were tested for HIV in a Voluntary Counseling Center and referred for TB screening in Cambodia (clinical, Southeast Asia, 3 LJ).	504	20/123 (16.3)	20/123 (16.3)
Total		29,523	557/9,626 (5.8)	495/8,148 (6.1)

a The following patients were excluded: (1) patients who were on TB treatment or prophylaxis at enrolment; (2) patients who were smear positive, but culture grew non- tuberculosis mycobacterium (NTM); and (3) patients who were smear positive, but culture negative.

b Patients were previously screened for TB before enrolment into the study.

LJ, Lowenstein-Jensen culture medium; MGIT, Mycobacterial Growth Indicator Tube culture system; PLHIV, persons living with human immunodeficiency virus; PLTB, persons with tuberculosis disease; VCT, voluntary counselling and testing for HIV.

Investigators for all included studies signed a data sharing and confidentiality agreement, and agreed to a data management, analysis, and publication plan. During design and analysis phases of the meta-analysis, the investigators of the studies and data managers of the included studies held multiple discussions by email, by teleconference, and in person in Geneva, Switzerland and Atlanta, Georgia, United States.

### Data Abstraction and Management

The list of variables from the most comprehensive dataset [20] was used to construct an initial master list of variables. All the variables from each study included in the meta-analysis were mapped to this master list. Principal investigators and data managers for the 12 studies worked with the meta-analysis investigators to ensure accurate mapping of data from the primary studies to the master variable list. In the end, the final dataset of the meta-analysis included 159 variables that appeared in at least two of the studies. We identified five symptoms common to most studies and limited the meta-analysis to the nine studies with substantially complete information for those five symptoms.

### Case Definitions

We defined a TB patient as any person living with HIV and at least one specimen culture positive for *Mycobacterium tuberculosis* (MTB). We defined participants as having no TB if cultures were negative for MTB and participants were judged not to have TB on the basis of the original study definition of the investigators. We excluded from the analysis: (1) patients who were receiving treatment for TB (infection or disease) at enrolment; (2) patients who were AFB smear positive, but whose culture grew non-tuberculous mycobacteria; and (3) patients who were AFB smear positive or scanty, but sputum culture negative.

### Sources of Study Heterogeneity

All studies were reviewed to identify study-level characteristics that could substantially impact the findings of the meta-analysis. Two studies were conducted exclusively among gold miners living in South Africa [21],[23], a population that may not be broadly generalizable, because of its demographics, its high prevalence of non-TB illnesses (e.g., silicosis) that can produce cough, and the practice of annual TB radiological screening. Five studies [18],[19],[22],[25],[27] were conducted among individuals drawn from a community setting through prevalence surveys, which may lead to enrolment of patients with a different spectrum of TB and HIV disease than would be found among patients seeking care. Three studies [19],[22],[23] involved participants who were previously screened for TB or who had had access to routine TB screening before being enrolled into the studies. Finally, three studies exclusively used liquid media to culture specimens [26]–[28], two studies used both solid and liquid media [18],[20], and seven studies exclusively used solid media (Table 1). Liquid media have substantially increased sensitivity for growing MTB, particularly in patients with low levels of TB bacilli, as would be expected in a population of people living with HIV being screened for TB [31]. Studies that used liquid media, therefore, would have improved ability to classify patients correctly into those who have TB and those who do not. We explored the impact of these factors on the performance of the screening algorithms and analyzed subsets of the final dataset grouped according to these characteristics.

### Data Analysis

We compared characteristics of patients with TB to those of patients without TB to derive a standardized rule for TB screening among people living with HIV. The goal of TB screening is to divide the population of people living with HIV into two groups: (1) those who do not have TB; and (2) those who need further evaluation for the diagnosis of TB (i.e., TB suspects). We restricted our analysis to clinical symptoms that could be readily assessed at any level of the health system and that were asked about in all studies: current cough (C), haemoptysis (H), fever (F), night sweats (S), and weight loss (W). Using the four studies that included chest radiography data [20],[23],[24],[26], we also evaluated the impact of adding abnormal chest radiography findings to the TB screening rule. Only observations with no missing data for the symptoms of interest were included in the analysis.

We considered “1-of-*n*” rules as candidates for screening for TB that could best classify patients into two groups (not TB and suspected TB) with high sensitivity [20]. The “1” represents the minimum number of symptoms that must be present in an individual to be classified as a suspected TB patient and the “*n*” represents the number of symptom(s) in a given rule. For example, a “1-of-3” rule could be a set of symptoms such as current cough, fever, and weight loss, abbreviated here as CFW. An individual with at least one symptom specified in this particular rule would be classified as a TB suspect; an individual without any of these symptoms would be classified as not having TB. We considered all combinations of the five candidate symptoms except for combinations that include both current cough and haemoptysis, yielding a total of 23 candidate rules: two 1-of-4 rules (CFSW, HFSW), seven 1-of-3 rules (CFS, CFW, CSW, HFS, HFW, HSW, FSW), nine 1-of-2 rules (CF, CS, CW, HF, HS, HW, FS, FW, SW), and five 1-of-1 rules (C, H, F, S, W) (see also Table 3.) The analysis with abnormal chest radiographic findings (X) considered these 23 rules, each augmented with this additional sign (e.g., CFSWX).

Other analyses have considered *m*-of-*n* rules with *m*>1 [20]. These rules cannot exceed the sensitivity of 1-of-*n* rules. Suppose that a positive screen requires the presence of at least two symptoms out of current cough, fever, and night sweats, the number of true positives for this 2-of-3 rule will not be greater than the number of true positives from the corresponding 1-of-3 rules. Because our aim in this analysis was to identify the most sensitive rule, we did not include rules of this kind in our analysis.

We applied two closely related methods for cross-study analysis of sensitivity and specificity of these 23 candidate screening rules: bivariate random-effects meta-analysis (BREMA) and the hierarchical summary relative operating characteristic (HSROC) curve [32],[33]. BREMA jointly models sensitivity and specificity while accommodating between-study heterogeneity, and HSROC models tradeoffs between sensitivity and specificity across study populations. Both methods can be unified in the same model. In addition to sensitivity and specificity, we calculated predictive value negative and likelihood ratio negative of each candidate rule [34]. Our goal was to identify a combination of symptoms that achieved the highest possible sensitivity and the lowest possible negative likelihood ratio for ruling out TB disease (without any predetermined cut-off points).

To further understand between-study heterogeneity and other factors associated with the diagnostic performance of the most sensitive rule, we analyzed several study-level predictors (setting, prior screening of study participants, culture medium used, and geographic region) and participant-level predictors (age, gender, CD4 T-lymphocyte cell count [CD4], and abnormal chest radiographic findings). Our analytic methods produced odds ratios that reflect the magnitude of association between each factor and the probability of correctly identifying persons with TB (sensitivity) or without TB (specificity). For a range of TB prevalence values, we calculated the negative predictive values at levels of each covariate. We calculated the ratio of the number of patients that screen positive but who actually have no TB (false positives) and hence unnecessarily require additional TB diagnostic evaluation (e.g., culture) to the number of patients that screen positive and actually have TB (true positives), which is referred to as the number needed to screen. We calculated this ratio for different rules using a theoretical population of 1,000 people living with HIV with different levels of TB prevalence. This ratio provides proxy information similar to a marginal cost-effectiveness analysis for different screening rules and it helps quantify how much a health program would need to invest (as measured by additional diagnostic tests) for every patient with TB identified through the screening rule [35].

Each observation with a missing covariate value was omitted from analysis of that covariate. BREMA models were fitted using SAS procedure Glimmix (SAS 9.22, SAS Institute), and further calculations were performed in R (R 2.10.1, R Development Core Team).

### Ethical Review

All data collection included in the meta-analysis was approved by institutional ethical review boards at the respective institutions during the original study; if necessary, principal investigators requested additional approval from institutional review boards for the inclusion of the primary dataset in the meta-analysis. All data for the meta-analysis were provided completely de-identified. In the meta-analysis dataset, investigators were not able to link case records to individuals.

## Results

**Figure 2:**
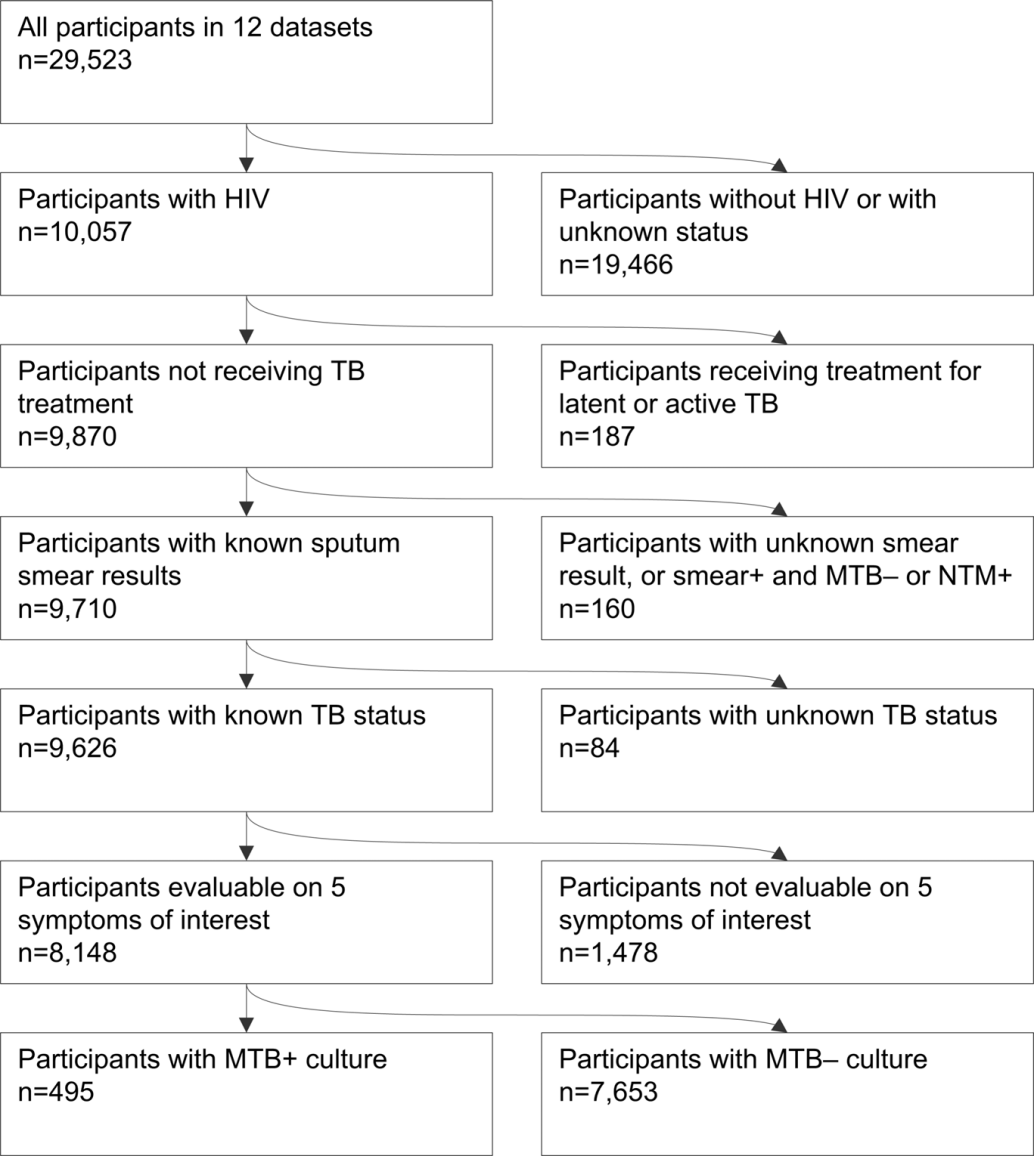
Flow chart of study participants included in the individual patient data meta-analysis.

Investigators provided data about 29,523 participants, of whom 10,057 were people living with HIV. The dataset included 9,626 people living with HIV who had TB screening and sputum culture performed, of whom 8,148 could be evaluated on the five symptoms of interest from nine of 12 studies (Figure 2).

Most patients (77% [7,386/9,626]) were from sub-Saharan Africa; the rest were from Southeast Asian countries. The median age was 34 y (interquartile range [IQR], 27–41 y). Of the 9,626 patients with HIV in the 12 studies, CD4 cell count information was available for 3,489 (36%) and chest radiography information for 3,903 (41%). The median CD4 count was 248 cells/µl (IQR, 107–409).

The overall prevalence of TB disease was 5.8% (557/9,626), ranging across studies from 0.4% to 25.7% (Table 1). More than half of TB patients (52% [288/557]) had sputum smear negative pulmonary TB, whereas 39% (218/557) had sputum smear positive pulmonary, and 5% (28/557) had exclusively extrapulmonary TB. The anatomic site of TB was not specified in 4% (23/557) of patients.

Table 2 summarizes the distribution of common variables, and Table S1 summarizes how each question was actually asked in each study. Because duration of cough was included in many studies but was asked about in different ways, we were able to analyze data using three different cough variables: cough in the past 4 wk (information available for 39.3% of participants); cough lasting for 2 wk or more (information available for 47.1%); and cough present in the last 24 h, which is referred to as “current cough” (information available for 89.6%).

**Table 2:** Characteristics of participants with and without TB for variables included in the analysis.

Characteristic	All PLHIV (*n = *9,626)		PLHIV with Data on the Five Symptoms (*n = *8,148)	
	No TB Disease (*n = *9,069), *n* (%)	TB Disease (*n = *557), *n* (%)	TB Disease (*n = *7,653), *n* (%)	TB Disease (*n = *495) *n* (%)
**Origin of patient**				
Sub-Saharan Africa	7,152 (78.9)	234 (42.0)	5,739 (75.0)	172 (34.8)
Southeast Asia	1,917 (21.1)	323 (58.0)	1,914 (25.0)	323 (65.2)
**Setting**				
Clinical	2,246 (24.8)	382 (68.5)	2,053 (26.8)	366 (73.9)
Community	5,322 (58.7)	125 (22.4)	5,058 (66.1)	111 (22.4)
Miners	1,501 (16.5)	50 (9.0)	542 (7.1)	18 (3.6)
**Sex**				
Male	4,957 (54.7)	356 (63.9)	3,811 (49.8)	309 (62.4)
Female	4,111 (45.3)	201 (36.1)	3,841 (50.2)	186 (37.6)
Missing or not recorded	1 (0.0)	0 (0.0)	1 (0.0)	0 (0.0)
**Median age (IQR), ** ***n = *** **8,633 (7,286)**	34 (27–41)	33 (28–40)	33 (27–40)	32 (28–39)
**Median CD4+ count (IQR), ** ***n = *** **3,489 (2,409)**	268 (126–427)	106 (38–241)	229 (94–391)	94 (33–215)
**Cough in the past 4 wk**				
Yes	1,439 (15.9)	303 (54.4)	1,270 (16.6)	278 (56.2)
No	1,909 (21.0)	129 (23.2)	1,067 (13.9)	110 (22.2)
Missing or not recorded	5,721 (63.1)	125 (22.4)	5,316 (69.5)	107 (21.6)
**Cough lasting for 2 wk or more**				
Yes	957 (10.6)	197 (35.4)	848 (11.1)	177 (35.8)
No	3,093 (34.1)	288 (51.7)	2,046 (26.7)	260 (52.5)
Missing or not recorded	5,019 (55.3)	72 (12.9)	4,759 (62.2)	58 (11.7)
**Haemoptysis**				
Yes	543 (6.0)	60 (10.8)	523 (6.8)	58 (11.7)
No	8,509 (71.4)	495 (88.9)	7,130 (93.2)	437 (88.3)
Missing or not recorded	17 (0.2)	2 (0.4)	0 (0.0)	0 (0.0)
**Current cough or cough in the past 24 h**				
Yes	1,625 (17.9)	274 (49.2)	1,530 (20.0)	260 (52.5)
No	6,474 (71.4)	250 (44.9)	6,123 (80.0)	235 (47.5)
Missing or not recorded	970 (10.7)	33 (5.9)	0 (0.0)	0 (0.0)
**Current fever or fever in the past 4 wk**				
Yes	1,801 (19.9)	294 (52.8)	1,669 (21.8)	280 (56.6)
No	7,002 (77.2)	246 (44.2)	5,984 (78.2)	215 (43.4)
Missing or not recorded	266 (2.9)	17 (3.0)	0 (0.0)	0 (0.0)
**Current night sweats or night sweats in the past 4 wk**				
Yes	1,710 (18.9)	242 (43.4)	1,497 (19.6)	225 (45.4)
No	7,329 (80.8)	313 (56.2)	6,156 (80.4)	270 (54.6)
Missing or not recorded	30 (0.3)	2 (0.4)	0 (0.0)	0 (0.0)
**Current weight loss or weight loss in the past 4 wk**				
Yes	2,434 (26.8)	333 (59.8)	2,258 (29.5)	308 (62.2)
No	6,478 (71.4)	218 (39.1)	5,395 (70.5)	187 (37.8)
Missing or not recorded	157 (1.7)	6 (1.1)	0 (0.0)	0 (0.0)
**Abnormal chest radiography**				
Yes	581 (6.4)	271 (48.7)	294 (3.8)	239 (48.3)
No	2,900 (32.0)	151 (27.1)	2,155 (28.1)	145 (29.3)
Missing or not recorded	5,588 (61.6)	135 (24.2)	5,204 (68.0)	111 (22.4)
**Abnormal chest radiography consistent with TB**				
Yes	377 (4.2)	227 (40.8)	261 (3.4)	209 (42.2)
No	2,589 (28.5)	144 (25.8)	1,641 (21.4)	129 (26.1)
Missing or not recorded	6,103 (67.3)	186 (33.4)	5,751 (75.2)	157 (31.7)
**Any one of current cough, fever, night sweats, or weight loss**				
Yes	3,591 (39.6)	425 (76.3)	3,563 (46.6)	418 (84.4)
No	4,090 (45.1)	77 (13.8)	4,090 (53.4)	77 (15.6)
Not evaluable	1,388 (15.3)	55 (9.9)	0 (0.0)	0 (0.0)

We analyzed the performance of individual and combinations of symptoms as screening rules using data from the 8,148 participants who could be evaluated based on the five candidate symptoms. Table 3 shows the diagnostic performance characteristics for the 23 candidate combinations of symptoms, sorted from highest sensitivity to lowest. The most sensitive rule was the presence of any one of the following symptoms: current cough, fever, night sweats, and weight loss (CFSW). The population-average sensitivity of this symptom combination was 78.9% (95% confidence interval [CI] 58.3%–90.9%) with the negative likelihood ratio of 0.426 (95% CI 0.349–0.520), which corresponds to a postscreening reduction in the probability of TB by 15%–20% [34].

**Table 3:** Diagnostic performance of 23 candidate 1-of-*n* rules.

Rule	Sensitivity (95% CI)		Specificity (95% CI)		LRN (95% CI)	
CFSW	78.9	(58.3–90.9)	49.6	(29.2–70.1)	0.426	(0.349–0.520)
HFSW	75.7	(53.9–89.2)^a^	52.7	(31.8–72.7)	0.461	(0.391–0.544)
CFW	74.0	(51.7–88.3)^a^	53.8	(32.8–73.6)	0.483	(0.416–0.561)
CSW	73.4	(51.0–88.0)	53.8	(32.8–73.5)	0.494	(0.428–0.570)
CFS	73.1	(50.6–87.9)	61.1	(39.7–79.0)	0.440	(0.382–0.506)
HFW	70.6	(47.5–86.4)	57.5	(36.2–76.4)	0.511	(0.454–0.576)
FSW	69.2	(45.9–85.6)	55.7	(34.5–75.0)	0.554	(0.497–0.617)
HSW	68.1	(44.6–85.0)	58.7	(37.3–77.2)	0.544	(0.492–0.602)
CW	65.3	(41.6–83.3)	60.3	(38.8–78.4)	0.576	(0.530–0.625)
CF	65.0	(41.3–83.1)	68.6	(47.7–83.9)	0.510	(0.470–0.553)
HFS	63.7	(39.9–82.3)	66.3	(45.2–82.4)	0.548	(0.509–0.589)
FW	63.1	(39.3–81.9)	61.4	(40.0–79.1)	0.601	(0.560–0.644)
SW	61.0	(37.2–80.5)	61.9	(40.5–79.5)	0.630	(0.594–0.669)
CS	59.7	(35.9–79.6)	69.4	(48.7–84.4)	0.581	(0.551–0.613)
HW	56.8	(33.3–77.6)	66.8	(45.7–82.8)	0.647	(0.620–0.675)
FS	56.3	(32.8–77.3)	70.1	(49.6–84.9)	0.623	(0.598–0.649)
HF	52.0	(29.2–74.1)	75.0	(55.6–87.7)	0.640	(0.620–0.660)
W	49.3	(27.0–71.9)	71.1	(50.8–85.5)	0.712	(0.693–0.733)
F	42.8	(22.2–66.3)	79.8	(62.4–90.4)	0.716	(0.695–0.738)
HS	38.9	(19.5–62.6)	78.1	(59.9–89.5)	0.782	(0.753–0.813)
C	38.5	(19.2–62.2)	81.8	(65.3–91.5)	0.753	(0.724–0.783)
S	31.4	(14.8–54.6)	82.2	(65.9–91.7)	0.835	(0.780–0.893)
H	5.9	(2.3–14.5)	94.4	(87.6–97.6)	0.996	(0.735–1.351)

Sensitivity, specificity, and likelihood ratio negative (LRN) from the bivariate random-effects meta-analysis model. Rule is at least one of the indicated symptoms. C, current cough; H, haemoptysis; F, fever; S, sweats; W, weight loss.

a *p*-Value >0.05 for the same sensitivity of the CFSW rule and the indicated rule. All specificities are significantly different from that of the CFSW rule.

LRN, likelihood ratio negative.

**Figure 3:**
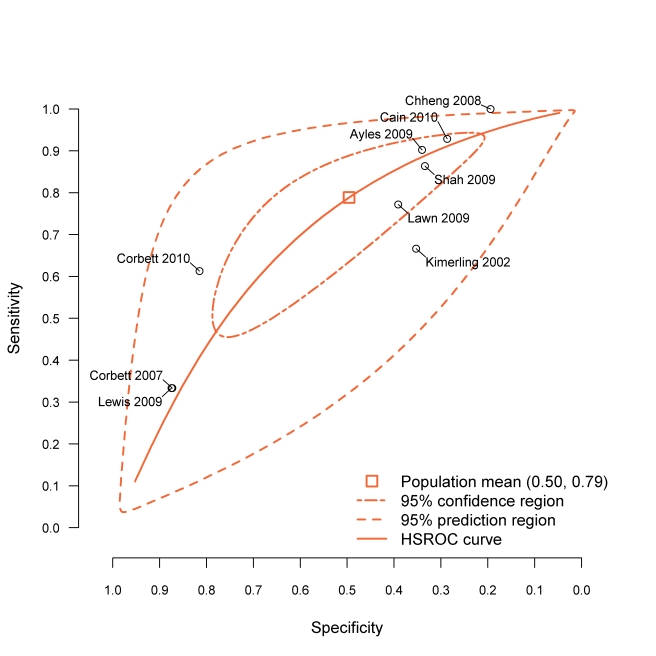
Diagnostic performance of CFSW rule in the included studies. BREMA, bivariate random-effects meta-analysis; HSROC, hierarchical summary relative operating characteristic.

The nine included studies demonstrated significant between-study heterogeneity on both sensitivity (*p*<0.001) and specificity (*p*<0.001) of the rule CFSW (see also Figure 3). The bivariate graphic shows that six studies have study-level specificities below and three above the population average specificity. Furthermore, this rule has the highest-ranking sensitivity in eight of the nine included studies (Table S2). The hierarchical summary relative operating characteristic curves (Figure S1) show slightly better overall diagnostic performance of the rules CFS and CF, but our application requires the highest sensitivity possible, allowing for some tradeoffs with lower specificity. Figure 3 shows that three studies are outliers, and they represent studies of patients who were previously screened for TB or studies in which much of the population likely had previous TB screening (e.g., miners); this can modify the performance characteristics of the screening rule.

The CFSW rule has sensitivity of 90.1% (95% CI 76.3%–96.2%) and 67.1% (95% CI 41.7%–85.3%) among participants selected from clinical and community settings, respectively. Similarly the sensitivity of the rule among those who had not been previously screened for TB was higher at 88.0% (95% CI 76.1%–94.4%) compared to those who had been screened for TB at 40.5% (95% CI 16.6%–69.9%). At the 95% confidence level, the sensitivity of this rule could not be statistically distinguished from the sensitivity of the rule that substitutes haemoptysis for current cough (HFSW, 75.7% sensitive [95% CI 53.9–89.2%]) or the rule that drops night sweats (CFW, 74.0% sensitive [51.7–88.3%]). All other rules had lower sensitivity.

Regression analysis of study-level predictors revealed that studies in which TB screening was performed in clinical settings had 4.5 times the odds for a true-positive screening result compared to studies in which TB screening was performed in a community setting (95% CI 1.0–19.5). Studies of participants who had not previously been screened for TB had 10.8 times the odds for a true-positive screen (95% CI 2.4–47.8) compared with studies in which participants had previously been screened for TB. Participants with CD4 cell count <200 cells/µl had 6.4 times the odds of a true-positive screen (95% CI 2.9–14.2). Statistically significant predictors of true-negative results include prescreening, geographic region, participant age ≥33 y, CD4 cell count <200 cells/ml, and abnormal result on chest radiograph (Table 4).

**Table 4:** Association of study-level and individual-level predictors with the diagnostic performance of CFSW rule.

Predictors		Sensitivity (95% CI)	Specificity (95% CI)
**Study level**			
Setting	Community	1.0	
	Clinical	4.45 (1.02, 19.46)^a^	0.25 (0.06–1.01)
	Miners	0.25 (0.02–2.51)	4.07 (0.44–37.68)
Screening	Prescreened for TB	1.0	
	Not screened for TB	10.82 (2.45–47.78)^a^	0.08 (0.06–0.12)^a^
Culture medium	Solid	1.0	
	Liquid	3.41 (0.57–20.30)	0.33 (0.06–1.97)
Region	Sub-Saharan Africa	1.0	
	Southeast Asia	4.03 (0.65–24.84)	0.20 (0.04–1.00)^a^
**Individual level**			
Age^b^	<33 y	1.0	
	≥33 y	1.43 (0.81–2.52)	0.74 (0.66–0.84)^a^
Gender	Female	1.0	
	Male	1.26 (0.71–2.24)	1.04 (0.93–1.16)
CD4 cell count^c^	≥200 cells/µl	1.0	
	<200 cells/µl	6.38 (2.87–14.17)^a^	0.46 (0.38–0.57)^a^
Abnormal chest radiograph^d^	No	1.0	
	Yes	1.36 (0.68–2.73)	0.41 (0.30–0.57)^a^

Values in each cell indicate the odds ratio for sensitivity or specificity compared with a referent group.

a *p*-value <0.05 for null hypothesis that odds ratio  = 1.

b Excludes Shah et al. [24].

c Includes only studies Cain et al. [20], Shah et al. [24], Lawn et al. [26], and Chheng et al. [29].

d Includes only studies Cain et al. [20], Lewis et al. [23], Shah et al. [24], and Lawn et al. [26].

Table 5 shows the negative predictive value and the numbers needed to screen for the CFSW rule adjusted for individual- and study-level covariates. In a setting with 5% TB prevalence among people living with HIV, the rule has a negative predictive value of 98.3% (95% CI 97.5%–98.8%) for patients screened in a clinical setting and 97.3% (95% CI 96.9%–97.7%) for patients screened in a community setting. The numbers needed to screen at the same prevalence of TB are 15 and 11 for clinical and community setting, respectively. The negative predictive value was similar in those having high (≥200) and low (<200) CD4 count at 96.9% (95% CI 95.1%–98.0%) and 98.9% (95% CI 97.5%–99.5%), respectively (see also Table S3).

**Table 5:** Negative predictive value (NPV) and number needed to screen (NNS) using rule CFSW in a hypothetical population of 1,000 people living with HIV stratified by study and individual level predictors.

Participants		1% TB Prevalence			5% TB Prevalence			10% TB Prevalence			20% TB Prevalence		
		NPV	95% CI	NNS	NPV	95% CI	NNS	NPV	95% CI	NNS	NPV	95% CI	NNS
**All study participants**		99.6	(99.5–99.6)	62	97.7	(97.4–98.0)	12	95.3	(94.6–95.9)	6	90.0	(88.6–91.3)	3
**All study participants excluding miners**		99.6	(99.5–99.7)	67	97.9	(97.5–98.2)	13	95.6	(94.8–96.3)	7	90.6	(89.0–92.1)	3
**Setting**	Clinical	99.7	(99.5–99.8)	78	98.3	(97.5–98.8)	15	96.4	(94.8–97.5)	8	92.3	(89.0–94.6)	4
	Community	99.5	(99.4–99.5)	55	97.3	(96.9–97.7)	11	94.5	(93.7–95.2)	5	88.5	(86.9–89.9)	3
	Miners	99.2	(98.7–99.5)	38	96.1	(93.8–97.6)	8	92.2	(87.8–95.1)	4	84.0	(76.1–89.6)	2
**Screening**	Nonscreened for TB	99.6	(99.5–99.7)	77	98.1	(97.5–98.5)	15	96.0	(94.9–96.9)	7	91.5	(89.2–93.3)	4
	Prescreened for TB	99.3	(99.3–99.3)	36	96.5	(96.2–96.7)	7	92.8	(92.4–93.2)	4	85.1	(84.3–86.0)	2
**Culture medium**	Liquid	99.6	(99.3–99.8)	75	98.2	(96.7–99.0)	15	96.2	(93.2–98.0)	7	91.9	(85.9–95.5)	3
	Solid	99.5	(99.4–99.5)	57	97.3	(97.0–97.7)	11	94.6	(93.8–95.2)	6	88.5	(87.1–89.8)	3
**Geography**	Southeast Asia	99.6	(99.2–99.8)	81	98,0	(95.9–99.0)	16	95,9	(91.6–98.0)	8	91.2	(83.0–95.6)	4
	Sub-Saharan Africa	99.5	(99.4–99.6)	52	97.4	(97.1–97.8)	10	94.8	(94.0–95.4)	5	88.9	(87.5–90.2)	3
**Age**	≥33 y	99.6	(99.5–99.7)	63	97.8	(97.2–98.2)	12	95.4	(94.3–96.4)	6	90.3	(88.0–92.1)	3
	<33 y	99.5	(99.4–99.6)	59	97.5	(97.0–97.9)	12	94.8	(94.0–95.6)	6	89.1	(87.4–90.6)	3
**Gender**	Male	99.5	(99.4–99.6)	64	97.5	(97.2–97.9)	13	95.0	(94.2–95.6)	6	89.3	(87.8–90.6)	3
	Female	99.6	(99.5–99.7)	60	98.0	(97.5–98.4)	12	95.8	(94.9,96.6)	6	91.0	(89.2–92.6)	3
**CD4 cell count**	≥200 cells/µl	99.4	(99.0–99.6)	80	96.9	(95.1–98.0)	16	93.6	(90.2–95.9)	8	86.7	(80.4–91.2)	4
	<200 cells/µl	99.8	(99.5–99.9)	81	98.9	(97.5–99.5)	16	97.8	(94.8–99.1)	8	95.1	(89.1–97.9)	4
**Abnormal chest radiograph**	Yes	99.4	(99.0–99.6)	83	97.0	(95.2–98.2)	16	93.9	(90.3–96.2)	8	87.2	(80.6–91.8)	4
	No	99.5	(99.3–99.7)	61	97.7	(96.7–98.4)	12	95.2	(93.2–96.7)	6	89.9	(85.9–92.9)	3

Four studies [20],[23],[24],[26] consistently recorded information on chest radiograph, allowing screening rules with this sign to be evaluated using data from 2,805 participants The addition of abnormal chest radiographic findings into the CFSW rule increases the sensitivity to 90.6% (95% CI 66.7%–97.9%) with a specificity of 38.9% (95% CI 12.8%–73.3%), and a likelihood ratio negative of 0.242 (95% CI 0.102–0.571). Fifteen of the 23 rules included in our analysis outperform the symptom-based CFSW rule when abnormal chest radiographic findings are added (Table S4).

**Figure 4:**
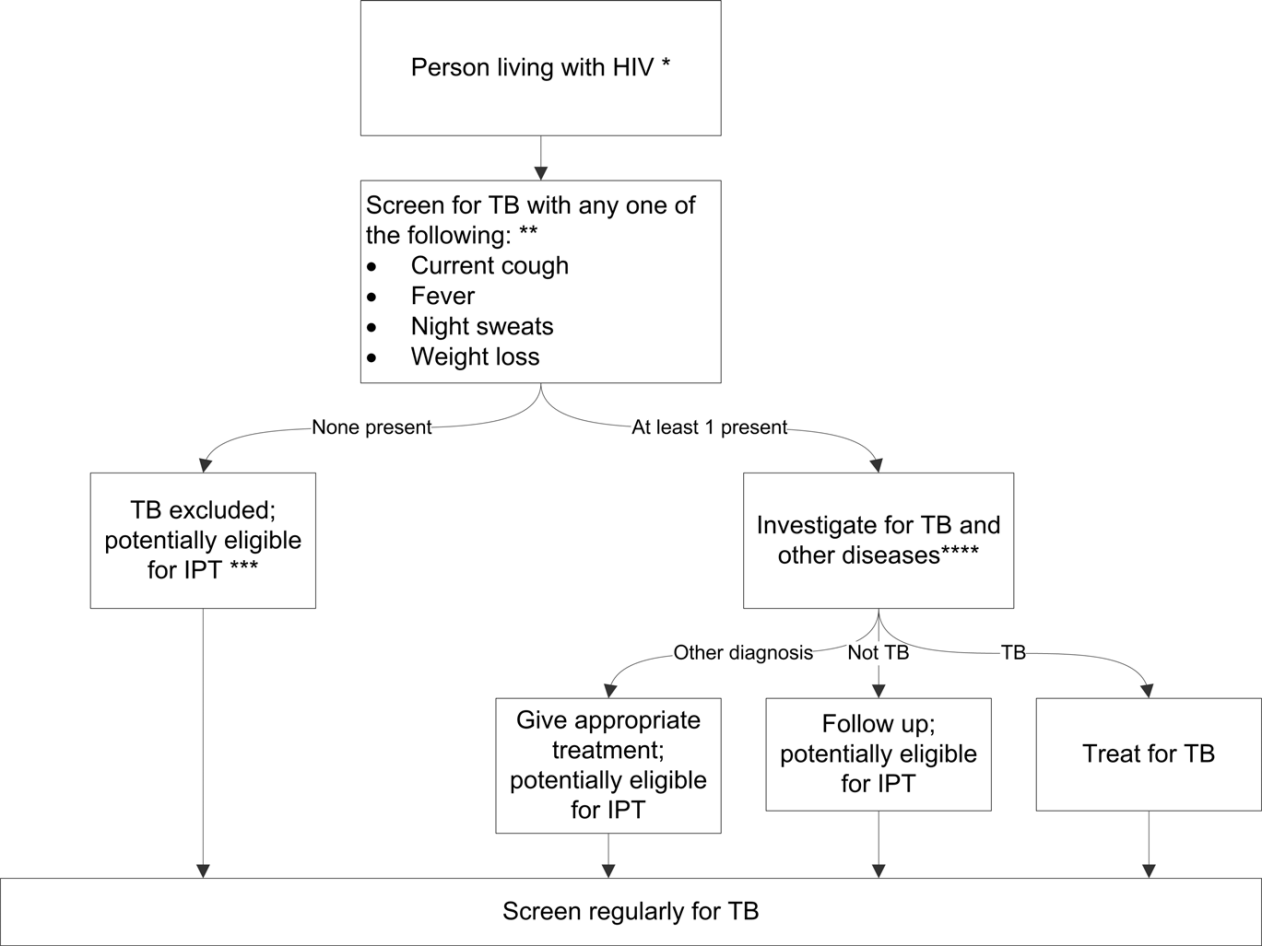
Algorithm for TB screening in person living with HIV in HIV prevalent and resource-constrained settings. * Every person living with HIV needs to be evaluated for ART eligibility, and all settings providing care should reduce TB transmission through proper measures. ** Chest radiography is not required to classify patients into the TB and not-TB groups, but can be done, if available, to increase the sensitivity of screening. *** Assess for contraindications, including active hepatitis (acute or chronic), regular and heavy alcohol consumption, and symptoms of peripheral neuropathy, is required prior to initiating IPT. Past history of TB is not a contraindication for starting IPT. Tuberculin skin test may be performed as part of eligibility screening in some settings. **** Investigations for TB should be done in accordance with existing national guidelines.

On the basis of our meta-analysis findings and incorporating current WHO recommendations on provision of IPT, we developed a simple TB screening algorithm for public health programmes to screen people living with HIV, and, depending on the outcome of screening, to either provide IPT or evaluate patients further for TB or other diseases (Figure 4).

## Discussion

We found that the absence of all of current cough, fever, night sweats, and weight loss can identify a subset of people living with HIV who have low probability of having TB disease. This screening rule was superior over other candidate rules in eight of the nine studies included and had an overall favourable performance over competing rules in the hierarchical summary relative operating characteristic (HSROC) analysis. We also demonstrated that the negative predictive value of the rule was high across a range of TB disease prevalence estimates and across different population subsets, including those with low and high CD4 count, and those drawn from clinical and community settings and South African miners. We believe that these screening questions are likely to be acceptable to practitioners, because they are symptoms classically associated with TB disease.

Underdiagnosis and delayed diagnosis of TB contribute to excess mortality among people living with HIV [17]. Similarly, concerns about the ability to reliably rule out active TB before initiating IPT have been a major barrier for wider use of this intervention. In the absence of a rapid and effective TB diagnostic tool available at the point-of-care, simple clinical algorithms must be used to screen people living with HIV for TB, dividing them into those in whom active TB is excluded and those who require further evaluation. This meta-analysis synthesizes the best available evidence for how to do this by relying on individual patient data of culture-confirmed TB cases from people living with HIV in the two regions of the world with the most severe burden of the TB and HIV dual epidemic.

The major change to existing practice would be the replacement of chronic cough with current cough as a screening question and the addition of other symptoms to standard screening. National TB programs have traditionally defined a TB “suspect” as someone with cough lasting greater than 2 or 3 wk, and designed case-finding activities to investigate up to ten TB suspects for every TB case detected [36]. However, studies included in this analysis have shown that chronic cough is highly insensitive for TB disease in people living with HIV; using this symptom as a screening rule would miss cases and contribute to diagnostic delays [16],[20]. Using the combination of symptoms that we propose, in a population of people living with HIV with a 5% TB prevalence (excluding miners), requires the investigation of 13 extra patients for every TB case detected, a ratio of TB suspects to a TB case not much different from what national TB control programmes target in the general population.

There has been ongoing debate about the importance of chest radiography in screening people living with HIV for IPT eligibility [37],[38]. Our analysis showed that the addition of abnormal chest radiography findings into the screening rule of CFSW increases the sensitivity of the rule by 11.7% (90.6% versus 78.9%) with a reduction of specificity by 10.7% (49.6% versus 38.9%). However, for example at a 5% TB prevalence rate among people living with HIV, augmenting the CFSW rule with abnormal chest radiographic findings increases the negative predictive value by a margin of only 1% (98.7% versus 97.8%), albeit with the same number of cases needed to be screened. On the other hand, the addition of abnormal chest radiographic findings to the rule at TB prevalence of 20% among people living with HIV increases the negative predictive value by almost 4% (94.3% versus 90.4%) without additional cases needed to be screened. It is also worth noting that the CFSW screening rule has higher sensitivity among those who presented into a clinical setting (90%) and among those who have not been previously screened for TB (88%).

Our findings show that the utility of the proposed symptom-based screening rule have excellent performance in most settings with TB and HIV burden. However, the negative predictive value will fall in those settings with higher TB prevalence when symptom screening alone is used, as it depends on prevalence of disease. In particular settings (e.g., antiretroviral clinics with a very high TB burden [39]), consideration must be given to use of an algorithm that contains chest radiography, or even adding additional sensitive investigations (e.g., culture) while screening people living with HIV for TB [30],[40]. People living with HIV and receiving IPT should also be regularly screened for TB during their visit to a health facility or contact with health care provider so as to promptly detect active TB, if it develops. Programme managers need to weigh the financial, technical, and logistic difficulties, and patient cost and inconvenience associated with performing chest radiography or other additional sensitive investigations on all people living with HIV as part of a screening program compared with an approach that relies only on symptomatic screening.

When interpreting our results, one must bear in mind that only a few variables were common to all studies included in the meta-analysis. It is possible that the addition of one or more symptoms not included in our list of common symptoms could have improved the performance of our proposed screening rule. However, at least one study included in our meta-analysis explored over 80 million combinations of about 100 signs and symptoms and found a symptom combination (cough and fever of any duration and night sweats for 3 weeks or longer), which was similar to the one we propose as the best performing one [20]. Furthermore, questions were not asked in a uniform manner across all studies, and the reporting of symptoms can be highly dependent on factors such as the quality of the interview and interviewer, the circumstances under which questions are asked, and the social and cultural factors that shape individual perceptions of symptoms and disease [41]. We reviewed all questions carefully with principal investigators and data managers to ensure accurate mapping of differently phrased questions to common variables. Our study relied on patients drawn from multiple countries and multiple settings, and the variation in the performance of the proposed screening rule across these different settings suggests that variation in patient self-report of symptoms is unlikely to have major impact, at least at the population level. In some studies, only one sputum specimen was collected for culture, while multiple cultures are required to maximize sensitivity. Some patients with TB may have been incorrectly classified as not having TB. Extrapulmonary TB is an important cause of morbidity and mortality in people living with HIV, but most studies included in the meta-analysis focused on screening for pulmonary TB. Young children were not included in the studies. We did not specifically look into the role of tuberculin skin test in the proposed screening rule. Ideally, the utility of the algorithm we propose, based on the screening rule from our meta-analysis, should be studied prospectively using a standardized protocol in multiple diverse sites; this is particularly important as the studies included in our analysis came from only two geographical regions of the world. Similarly, because of the time required for the data aggregation, statistical analysis, manuscript preparation, and publication, there was one potentially eligible study that was not included in our analysis [30]. We believe that the exclusion of this single study from South Africa, a country from which we have included similar studies already, will not affect the interpretation of our data and conclusions. In the future, as more studies are reported, particularly from other regions, it will be important to repeat the meta-analysis.

Greatly improving TB screening, diagnosis, and treatment in people living with HIV will require deployment of a rapid, accurate, point-of-care TB diagnostic test. In the absence of such a test, we believe that a standardized algorithm employing symptoms, as we propose here, can improve the diagnosis and treatment of TB for people living with HIV, and by doing so would save many lives. Reliable exclusion of TB disease will facilitate safer initiation of antiretroviral therapy and will allow for broader use of IPT, which can substantially reduce TB incidence. Earlier and accurate HIV and TB screening and treatment may also help identify infectious cases earlier, thereby reducing both HIV and TB transmission. Evidence-based and internationally recommended guidelines should be used to expedite the diagnosis and treatment of TB in people living with HIV [42].

## Supporting Information

Figure S1 Hierarchical summary relative operating characteristic (HSROC) curves for the 23 candidate 1-of-*n* rules.
(0.08 MB DOC)

Table S1 Phrasing of questions that were used in all studies to ask about five common symptoms.
(0.09 MB DOC)

Table S2 Study-specific values and rankings of the sensitivity of each candidate screening rule in the nine studies included.
(0.14 MB DOC)

Table S3 Diagnostic performance of all 23 candidate rules and number needed to screen in a hypothetical population of 1,000 people living with HIV stratified by TB prevalence among people living with HIV.
(0.15 MB DOC)

Table S4 Diagnostic performance of 23 candidate rules that include abnormal chest radiograph and number needed to screen in a hypothetical population of 1,000 people living with HIV stratified by TB prevalence among people living with HIV.
(0.15 MB DOC)

## Abbreviations

AFB: acid-fast bacilli
ART: antiretroviral treatment
CD4: CD4 T-lymphocyte cell count
CI: confidence interval
IQR: interquartile range
IPT: isoniazid preventive therapy
TB: tuberculosis
