# Supplementary material for: Development of a Standardized Screening Rule for Tuberculosis in People Living with HIV in Resource-Constrained Settings: Individual Participant Data Meta-analysis of Observational Studies
Source: PLoS Med. 2011 Jan 18;8(1):e1000391. doi: 10.1371/journal.pmed.1000391 (PMC3022524; doi:10.1371/journal.pmed.1000391)
Supplement: Figure S1 — Hierarchical summary relative operating characteristic (HSROC) curves for the 23 candidate 1-of-n rules. (0.08 MB DOC) [file pmed.1000391.s001.doc]

**Figure S1: Hierarchical summary relative operating characteristic (HSROC) curves for the 23 candidate 1-of-*n* rules.**
