# Supplementary material for: Development of a Standardized Screening Rule for Tuberculosis in People Living with HIV in Resource-Constrained Settings: Individual Participant Data Meta-analysis of Observational Studies
Source: PLoS Med. 2011 Jan 18;8(1):e1000391. doi: 10.1371/journal.pmed.1000391 (PMC3022524; doi:10.1371/journal.pmed.1000391)
Supplement: Table S1 — Phrasing of questions that were used in all studies to ask about five common symptoms. (0.09 MB DOC) [file pmed.1000391.s002.doc]

**Table S1. Phrasing of questions that were used in all studies to ask about five common symptoms.**

| **Reference** | **Cough** | **Haemoptysis** | **Fever** | **Night sweats** | **Weight loss** |
| --- | --- | --- | --- | --- | --- |
| Ayles et al  2009 | Are you currently coughing? If yes how long have you been coughing for? (coded < 1 week, 1-3 weeks, >3 weeks, > 6 months) | Do you currently cough up blood? | Do you currently have fever? | Do you currently have sweating at night? | In the last month, did you lose weight? |
| Corbett et al  2010 | Do you cough at any time of the day or night even mildly? If you have a cough, is it getting worse? For how many weeks or months have you had a cough? Has cough been present for 2 weeks or more? | Have you coughed up blood at any time in the last year? | Do you have a hot body or fever? If yes, for how many weeks have you had hot body or fever? | Do you have night sweats? Do you have to change sheets or night clothes during the night? If yes, for how many weeks have you had night sweats? | Are you loosing weight? If yes, for how many weeks have you been losing weight? |
| Cain et al  2010 | In the four weeks before now, have you had cough? - Did you have a cough in the past 24 hours? | In the four weeks before now, have you coughed up blood? - Have you coughed up blood in the past 24 hours? | In the four weeks before now, have you had fever? - Did you have a fever in the past 24 hours? | In the four weeks before now, have you had drenching night sweats? - Did you have night sweats in the past 24 hours? | In the four weeks before now, have you had lost weight? |
| Day et al  2006 | Does the patient have a new or worsening cough? If the cough is getting worse, how many weeks has it been getting worse? | Does the patient have haemoptysis? | Does the patients have symptomatic fever? How many weeks have the patients been having fevers? | Does the patient have drenching night sweats? | Does the patient have recent weight loss (in the last 6 months)? |
| Corbett et al  2007 | Do you usually or often cough at any time of the day or night even midely? If yes, is the cough getting worse? For how many weeks or months have you had a cough? | Have you coughed up blood at any time in the last year? | Do you have a hot body or fever? if yes, for how many weeks or months have you had hot body or fever? | Do you have night sweats? If yes, for how many weeks or months have you had night sweats? Do you ever have to change sheets or night clothes because of sweat? | Have you lost weight or has your clothing become loose in the last 6 months? |
| Lewis et al  2009 | Have you got a new cough, or a cough that has been getting worse in the last month? | Have you been coughing up blood? | Have you had any fevers (getting hot and cold with shivering)? | Do you sweat so much at night that your cloths are soaking wet? | Have you found that you have lost weight in the last six months so that your cloths have become looser? |
| Shah et al  2009 | Do you have a cough now? If no cough now, have you had a cough in the last 2 months? | If you cough now, have you been coughing up blood? If no cough now, but been coughing in the last 2 months, were you coughing up blood? | In the 2 months before coming to the VCT clinic, what symptoms have you been having? - fever | In the 2 months before coming to the VCT clinic, what symptoms have you been having? - night sweats | In the 2 months before coming to the VCT clinic, what symptoms have you been having? - weight loss |
| Kimerling et al 2002 | Please tell me, do you have any of the following symptoms at this moment? - cough with or without sputum for more than 21 days | Please tell me, do you have any of the following symptoms at this moment? - cough with sputum with blood | Please tell me, do you have any of the following symptoms at this moment? – fever | Please tell me, do you have any of the following symptoms at this moment? - night sweats | Please tell me, do you have any of the following symptoms at this moment? - feel rapid weight loss |
| Lawn et al  2009 | Do you have a cough? How long have you been coughing? Have you been coughing for more than 2 weeks? [Variable scored according to whether the patient reported a current cough with duration of ≥2 weeks]. | Have you coughed up any blood recently? | Have you recently had fever? | Have you recently had any soaking night sweats? | Have you been rapidly losing weight recently? |
| Wood et al  2007 | Do you have a cough? If yes, how long have you had the cough (specify in days, weeks, or months) | Do you have a cough? If yes, have you coughed up blood in the last three weeks? | Not applicable | Are you having drenching night sweats? If yes, how long have you had night sweats (specify in days, weeks, or months) | Have you lost weight in the last months (do your pants or dresses feel looser on you)? |
| Mohammed et al 2004 | Have you had persistent cough ≥2 weeks? | Have you coughed up blood in past 4 weeks? | Have you had fever ≥2 weeks? | Have you had drenching night sweats in the past 2 weeks? | Have you lost weight in past 4 weeks? |
| Chheng et al  2008 | Please tell me, do you have any of the following symptoms at this moment? - cough with or without sputum for more than 21 days | Please tell me, do you have any of the following symptoms at this moment? - cough with sputum with blood | Please tell me, do you have any of the following symptoms at this moment? – fever | Please tell me, do you have any of the following symptoms at this moment? - night sweats | Please tell me, do you have any of the following symptoms at this moment? - feel rapid weight loss |
