# Supplementary material for: Development of a Standardized Screening Rule for Tuberculosis in People Living with HIV in Resource-Constrained Settings: Individual Participant Data Meta-analysis of Observational Studies
Source: PLoS Med. 2011 Jan 18;8(1):e1000391. doi: 10.1371/journal.pmed.1000391 (PMC3022524; doi:10.1371/journal.pmed.1000391)
Supplement: Table S2 — Study-specific values and rankings of the sensitivity of each candidate screening rule in the nine studies included. (0.14 MB DOC) [file pmed.1000391.s003.doc]

**Table S2**. Study-specific values and rankings of the sensitivity of each candidate screening rule in the nine studies included.

|  | **Ayles  *et al* 2009** | | **Corbett *et al* 2010** | | **Cain *et al* 2010** | | **Corbett *et al* 2007** | | **Lewis *et al***  **2009** | | **Shah *et al* 2009** | | **Kimerling *et al* 2002** | | **Lawn *etal* 2009** | | **Chheng *et al***  **2008** | |
| --- | --- | --- | --- | --- | --- | --- | --- | --- | --- | --- | --- | --- | --- | --- | --- | --- | --- | --- |
|  | **Value** | **Rank** | **Value** | **Rank** | **Value** | **Rank** | **Value** | **Rank** | **Value** | **Rank** | **Value** | **Rank** | **Value** | **Rank** | **Value** | **Rank** | **Value** | **Rank** |
| CFSW | 90.2 | 1 | 61.3 | 1 | 92.9 | 1 | 33.3 | 1 | 33.3 | 1 | 86.4 | 1 | 66.7 | 9 | 77.2 | 1 | 100.0 | 1 |
| HFSW | 78.0 | 6 | 61.3 | 1 | 88.0 | 4 | 0.0 | 9 | 27.8 | 5 | 86.4 | 1 | 97.2 | 1 | 73.7 | 2 | 100.0 | 1 |
| CFW | 82.9 | 4 | 61.3 | 1 | 91.8 | 2 | 33.3 | 1 | 16.7 | 13 | 81.8 | 8 | 55.6 | 12 | 71.9 | 5 | 100.0 | 1 |
| CSW | 90.2 | 1 | 61.3 | 1 | 87.3 | 7 | 33.3 | 1 | 33.3 | 1 | 86.4 | 1 | 61.1 | 10 | 73.7 | 2 | 100.0 | 1 |
| CFS | 85.4 | 3 | 58.1 | 7 | 90.3 | 3 | 33.3 | 1 | 33.3 | 1 | 86.4 | 1 | 61.1 | 10 | 63.2 | 9 | 100.0 | 1 |
| HFW | 70.7 | 11 | 61.3 | 1 | 86.5 | 8 | 0.0 | 9 | 11.1 | 17 | 77.3 | 12 | 97.2 | 1 | 63.2 | 9 | 100.0 | 1 |
| FSW | 78.0 | 6 | 51.6 | 12 | 87.6 | 6 | 0.0 | 9 | 27.8 | 5 | 86.4 | 1 | 44.4 | 15 | 73.7 | 2 | 100.0 | 1 |
| HSW | 73.2 | 10 | 58.1 | 7 | 80.5 | 11 | 0.0 | 9 | 27.8 | 5 | 81.8 | 8 | 97.2 | 1 | 68.4 | 6 | 100.0 | 1 |
| CW | 78.0 | 6 | 61.3 | 1 | 83.1 | 10 | 33.3 | 1 | 16.7 | 13 | 77.3 | 12 | 47.2 | 14 | 68.4 | 6 | 100.0 | 1 |
| CF | 75.6 | 9 | 54.8 | 10 | 88.0 | 4 | 33.3 | 1 | 16.7 | 13 | 81.8 | 8 | 44.4 | 15 | 49.1 | 17 | 100.0 | 1 |
| HFS | 68.3 | 14 | 45.2 | 16 | 80.1 | 12 | 0.0 | 9 | 27.8 | 5 | 86.4 | 1 | 97.2 | 1 | 50.9 | 15 | 100.0 | 1 |
| FW | 70.7 | 11 | 51.6 | 12 | 86.1 | 9 | 0.0 | 9 | 11.1 | 17 | 77.3 | 12 | 33.3 | 20 | 63.2 | 9 | 100.0 | 1 |
| SW | 70.7 | 11 | 48.4 | 15 | 80.1 | 12 | 0.0 | 9 | 27.8 | 5 | 81.8 | 8 | 38.9 | 17 | 68.4 | 6 | 100.0 | 1 |
| CS | 80.5 | 5 | 58.1 | 7 | 76.4 | 15 | 33.3 | 1 | 33.3 | 1 | 77.3 | 12 | 55.6 | 12 | 56.1 | 14 | 90.0 | 17 |
| HW | 56.1 | 18 | 54.8 | 10 | 73.4 | 18 | 0.0 | 9 | 11.1 | 17 | 68.2 | 18 | 94.4 | 7 | 57.9 | 12 | 90.0 | 17 |
| FS | 68.3 | 14 | 35.5 | 19 | 79.0 | 14 | 0.0 | 9 | 27.8 | 5 | 86.4 | 1 | 36.1 | 18 | 50.9 | 15 | 100.0 | 1 |
| HF | 48.8 | 21 | 35.5 | 19 | 75.3 | 16 | 0.0 | 9 | 0.0 | 21 | 72.7 | 16 | 97.2 | 1 | 28.1 | 21 | 100.0 | 1 |
| W | 53.7 | 20 | 45.2 | 16 | 73.0 | 19 | 0.0 | 9 | 11.1 | 17 | 68.2 | 18 | 25.0 | 22 | 57.9 | 12 | 90.0 | 17 |
| F | 48.8 | 21 | 25.8 | 21 | 73.8 | 17 | 0.0 | 9 | 0.0 | 21 | 72.7 | 16 | 13.9 | 23 | 24.6 | 22 | 100.0 | 1 |
| HS | 58.5 | 17 | 38.7 | 18 | 50.6 | 21 | 0.0 | 9 | 27.8 | 5 | 59.1 | 20 | 97.2 | 1 | 36.8 | 19 | 85.0 | 20 |
| C | 65.9 | 16 | 51.6 | 12 | 58.1 | 20 | 33.3 | 1 | 16.7 | 13 | 50.0 | 22 | 36.1 | 18 | 40.4 | 18 | 55.0 | 22 |
| S | 56.1 | 18 | 25.8 | 21 | 47.6 | 22 | 0.0 | 9 | 27.8 | 5 | 59.1 | 20 | 30.6 | 21 | 36.8 | 19 | 85.0 | 20 |
| H | 4.9 | 23 | 12.9 | 23 | 4.1 | 23 | 0.0 | 9 | 0.0 | 21 | 0.0 | 23 | 94.4 | 7 | 7.0 | 23 | 15.0 | 23 |
