# Supplementary material for: Development of a Standardized Screening Rule for Tuberculosis in People Living with HIV in Resource-Constrained Settings: Individual Participant Data Meta-analysis of Observational Studies
Source: PLoS Med. 2011 Jan 18;8(1):e1000391. doi: 10.1371/journal.pmed.1000391 (PMC3022524; doi:10.1371/journal.pmed.1000391)
Supplement: Table S3 — Diagnostic performance of all 23 candidate rules and number needed to screen in a hypothetical population of 1,000 people living with HIV stratified by TB prevalence among people living with HIV. (0.15 MB DOC) [file pmed.1000391.s004.doc]

**Table S3.** Diagnostic performance of all 23 candidate rules and number needed to screen in a hypothetical population of 10000 people living with HIV stratified by TB prevalence among people living with HIV.

|  |  |  |  |  |  |  | 1% TB Prevalence | | | 5% TB Prevalence | | | 10% TB Prevalence | | | 20% TB Prevalence | | |
| --- | --- | --- | --- | --- | --- | --- | --- | --- | --- | --- | --- | --- | --- | --- | --- | --- | --- | --- |
| Rule | Sensitivity (95% CI) | | Specificity (95% CI) | | LRN (95% CI) | | NPV | 95% CI | NNS | NPV | 95% CI | NNS | NPV | 95% CI | NNS | NPV | 95% CI | NNS |
| CFSW | 78.9 | (58.3, 90.9) | 49.6 | (29.2, 70.1) | 0.426 | (0.349, 0.520) | 99.6 | (99.5, 99.6) | 64 | 97.8 | (97.3, 98.2) | 13 | 95.5 | (94.5, 96.3) | 6 | 90.4 | (88.5, 92.0) | 3 |
| HFSW | 75.7 | (53.9, 89.2)* | 52.7 | (31.8, 72.7) | 0.461 | (0.391, 0.544) | 99.5 | (99.5, 99.6) | 62 | 97.6 | (97.2, 98.0) | 12 | 95.1 | (94.3, 95.8) | 6 | 89.7 | (88.0, 91.1) | 3 |
| CFW | 74.0 | (51.7, 88.3)* | 53.8 | (32.8, 73.6) | 0.483 | (0.416, 0.561) | 99.5 | (99.4, 99.6) | 62 | 97.5 | (97.1, 97.9) | 12 | 94.9 | (94.1, 95.6) | 6 | 89.2 | (87.7, 90.6) | 3 |
| CSW | 73.4 | (51.0, 88.0) | 53.8 | (32.8, 73.5) | 0.494 | (0.428, 0.570) | 99.5 | (99.4, 99.6) | 63 | 97.5 | (97.1, 97.8) | 12 | 94.8 | (94.0, 95.5) | 6 | 89.0 | (87.5, 90.3) | 3 |
| CFS | 73.1 | (50.6, 87.9) | 61.1 | (39.7, 79.0) | 0.440 | (0.382, 0.506) | 99.6 | (99.5, 99.6) | 53 | 97.7 | (97.4, 98.0) | 11 | 95.3 | (94.7, 95.9) | 5 | 90.1 | (88.8, 91.3) | 3 |
| HFW | 70.6 | (47.5, 86.4) | 57.5 | (36.2, 76.4) | 0.511 | (0.454, 0.576) | 99.5 | (99.4, 99.5) | 60 | 97.4 | (97.1, 97.7) | 12 | 94.6 | (94.0, 95.2) | 6 | 88.7 | (87.4, 89.8) | 3 |
| FSW | 69.2 | (45.9, 85.6) | 55.7 | (34.5, 75.0) | 0.554 | (0.497, 0.617) | 99.4 | (99.4, 99.5) | 64 | 97.2 | (96.9, 97.5) | 13 | 94.2 | (93.6, 94.8) | 6 | 87.8 | (86.6, 89.0) | 3 |
| HSW | 68.1 | (44.6, 85.0) | 58.7 | (37.3, 77.2) | 0.544 | (0.492, 0.602) | 99.5 | (99.4, 99.5) | 61 | 97.2 | (96.9, 97.5) | 12 | 94.3 | (93.7, 94.8) | 6 | 88.0 | (86.9, 89.0) | 3 |
| CW | 65.3 | (41.6, 83.3) | 60.3 | (38.8, 78.4) | 0.576 | (0.530, 0.625) | 99.4 | (99.4, 99.5) | 61 | 97.1 | (96.8, 97.3) | 12 | 94.0 | (93.5, 94.4) | 6 | 87.4 | (86.5, 88.3) | 3 |
| CF | 65.0 | (41.3, 83.1) | 68.6 | (47.7, 83.9) | 0.510 | (0.470, 0.553) | 99.5 | (99.4, 99.5) | 48 | 97.4 | (97.2, 97.6) | 10 | 94.6 | (94.2, 95.0) | 5 | 88.7 | (87.9, 89.5) | 2 |
| HFS | 63.7 | (39.9, 82.3) | 66.3 | (45.2, 82.4) | 0.548 | (0.509, 0.589) | 99.4 | (99.4, 99.5) | 53 | 97.2 | (97.0, 97.4) | 11 | 94.3 | (93.9, 94.6) | 5 | 88.0 | (87.2, 88.7) | 3 |
| FW | 63.1 | (39.3, 81.9) | 61.4 | (40.0, 79.1) | 0.601 | (0.560, 0.644) | 99.4 | (99.4, 99.4) | 61 | 96.9 | (96.7, 97.1) | 12 | 93.7 | (93.3, 94.1) | 6 | 86.9 | (86.1, 87.7) | 3 |
| SW | 61.0 | (37.2, 80.5) | 61.9 | (40.5, 79.5) | 0.630 | (0.594, 0.669) | 99.4 | (99.3, 99.4) | 62 | 96.8 | (96.6, 97.0) | 12 | 93.5 | (93.1, 93.8) | 6 | 86.4 | (85.7, 87.1) | 3 |
| CS | 59.7 | (35.9, 79.6) | 69.4 | (48.7, 84.4) | 0.581 | (0.551, 0.613) | 99.4 | (99.4, 99.4) | 51 | 97.0 | (96.9, 97.2) | 10 | 93.9 | (93.6, 94.2) | 5 | 87.3 | (86.7, 87.9) | 3 |
| HW | 56.8 | (33.3, 77.6) | 66.8 | (45.7, 82.8) | 0.647 | (0.620, 0.675) | 99.4 | (99.3, 99.4) | 58 | 96.7 | (96.6, 96.8) | 12 | 93.3 | (93.0, 93.6) | 6 | 86.1 | (85.6, 86.6) | 3 |
| FS | 56.3 | (32.8, 77.3) | 70.1 | (49.6, 84.9) | 0.623 | (0.598, 0.649) | 99.4 | (99.3, 99.4) | 53 | 96.8 | (96.7, 96.9) | 11 | 93.5 | (93.3, 93.8) | 5 | 86.5 | (86.0, 87.0) | 3 |
| HF | 52.0 | (29.2, 74.1) | 75.0 | (55.6, 87.7) | 0.640 | (0.620, 0.660) | 99.4 | (99.3, 99.4) | 48 | 96.7 | (96.6, 96.8) | 10 | 93.4 | (93.2, 93.6) | 5 | 86.2 | (85.8, 86.6) | 2 |
| W | 49.3 | (27.0, 71.9) | 71.1 | (50.8, 85.5) | 0.712 | (0.693, 0.733) | 99.3 | (99.3, 99.3) | 58 | 96.4 | (96.3, 96.5) | 12 | 92.7 | (92.5, 92.9) | 6 | 84.9 | (84.5, 85.2) | 3 |
| F | 42.8 | (22.2, 66.3) | 79.8 | (62.4, 90.4) | 0.716 | (0.695, 0.738) | 99.3 | (99.3, 99.3) | 47 | 96.4 | (96.3, 96.5) | 9 | 92.6 | (92.4, 92.8) | 5 | 84.8 | (84.4, 85.2) | 2 |
| HS | 38.9 | (19.5, 62.6) | 78.1 | (59.9, 89.5) | 0.782 | (0.753, 0.813) | 99.2 | (99.2, 99.2) | 56 | 96.0 | (95.9, 96.2) | 11 | 92.0 | (91.7, 92.3) | 6 | 83.6 | (83.1, 84.2) | 3 |
| C | 38.5 | (19.2, 62.2) | 81.8 | (65.3, 91.5) | 0.753 | (0.724, 0.783) | 99.2 | (99.2, 99.3) | 47 | 96.2 | (96.0, 96.3) | 10 | 92.3 | (92.0, 92.6) | 5 | 84.2 | (83.6, 84.7) | 2 |
| S | 31.4 | (14.8, 54.6) | 82.2 | (65.9, 91.7) | 0.835 | (0.780, 0.893) | 99.2 | (99.1, 99.2) | 57 | 95.8 | (95.5, 96.1) | 11 | 91.5 | (91.0, 92.0) | 6 | 82.7 | (81.7, 83.7) | 3 |
| H | 5.9 | (2.3, 14.5) | 94.4 | (87.6, 97.6) | 0.996 | (0.735, 1.351) | 99.0 | (98.7, 99.3) | 94 | 95.0 | (93.4, 96.3) | 18 | 90.0 | (87.0, 92.5) | 9 | 80.1 | (74.8, 84.5) | 4 |

LRN: Likelihood ratio negative. NNS: Number needed to screen NPV: Negative predictive value

Rule is at least one of the indicated symptoms. C: current cough, H: hemoptysis, F: fever, S: sweats, W: weight loss

*P*-value > 0.05 for the same accuracy of CFSW rule and indicated rule.

**Explanation for tables S3 and S4 and number needed to screen:** Program managers in resource constrained will need to balance the additional resources (e.g. culture and other TB investigations) needed with the sensitivity gained while applying the different rules. Our analysis attempted to measure this balance using the ratio of the number of patients that screen positive but who actually have no TB (false positives)and hence unnecessarily require additional TB diagnostic evaluation (e.g culture) to the number of patients that screen positive and actually have TB (true positives) . The addition of abnormal chest radiograph findings into the screening rule of CFSW increases the sensitivity of the rule by 11.7% (90.6% vs.78.9%) with a reduction of specificity by 10.7% (49.6 % vs.38.9%). However, for example at a 5% TB prevalence rate among people living with HIV, augmenting the CFSW rule with abnormal chest radiographic findings increases the negative predictive value by a margin of only 1% (98.7% vs. 97.8%). Albeit with the same number of cases needed to be screened. On the other hand, the addition of abnormal chest radiographic findings to the rule at TB prevalence of 20% among people living with HIV increases the negative predictive value by almost 4% (94.3% vs. 90.4%) without additional cases needed to be screened. This help to define the capacity of the health program and the acceptability of the additional resource needed to avoid the missing of a single case of TB during the screening process.
